# Revisiting the Role of Astrocytic MAOB in Parkinson’s Disease

**DOI:** 10.3390/ijms23084453

**Published:** 2022-04-18

**Authors:** Min-Ho Nam, Moonsun Sa, Yeon Ha Ju, Mingu Gordon Park, C. Justin Lee

**Affiliations:** 1 Brain Science Institute, Korea Institute of Science and Technology, Seoul 02792, Korea; yeonha92@kist.re.kr; 2 Department of KHU-KIST Convergence Science and Technology, Kyung Hee University, Seoul 02453, Korea; 3 KU-KIST Graduate School of Converging Science and Technology, Korea University, Seoul 02841, Korea; moonsun@ibs.re.kr (M.S.); mgpark@ibs.re.kr (M.G.P.); 4 Center for Cognition and Sociality, Institute for Basic Science, Daejeon 34126, Korea

**Keywords:** astrocyte, Parkinson’s disease, MAOB, GABA, H_2_O_2_, dopamine

## Abstract

Monoamine oxidase-B (MAOB) has been believed to mediate the degradation of monoamine neurotransmitters such as dopamine. However, this traditional belief has been challenged by demonstrating that it is not MAOB but MAOA which mediates dopamine degradation. Instead, MAOB mediates the aberrant synthesis of GABA and hydrogen peroxide (H_2_O_2_) in reactive astrocytes of Parkinson’s disease (PD). Astrocytic GABA tonically suppresses the dopaminergic neuronal activity, whereas H_2_O_2_ aggravates astrocytic reactivity and dopaminergic neuronal death. Recently discovered reversible MAOB inhibitors reduce reactive astrogliosis and restore dopaminergic neuronal activity to alleviate PD symptoms in rodents. In this perspective, we redefine the role of MAOB for the aberrant suppression and deterioration of dopaminergic neurons through excessive GABA and H_2_O_2_ synthesis of reactive astrocytes in PD.

## 1. MAOB Expression in the Brain

Monoamine oxidase B (MAOB) is a class of monoamine oxidase (MAO), a family of enzymes that catalyzes the oxidation of monoamines at the outer membrane of mitochondria. MAO was first discovered in the liver and named tyramine oxidase by Mary Bernheim in 1928 [1]. After a decade, it was renamed MAO to distinguish it from oxidatively deaminated diamines. In the 1950s, this enzyme was identified in the brain and discovered to oxidize catecholamines, such as dopamine (DA), adrenaline, and noradrenaline [2,3,4]. Nowadays, MAO is believed to maintain the homeostasis of monoamine neurotransmitters and metabolites in the brain [5].

MAOB is expressed throughout the whole brain, including the cerebral cortex, cerebellum, hippocampus, and midbrain [6,7]. Among several types of brain cells, a series of previous immunohistochemical and in situ hybridization studies demonstrated that MAOB is preferentially placed in astrocytes of both rodent and human brains [8,9,10]. In addition to astrocytes, several reports demonstrated that MAOB is also expressed in serotoninergic and histaminergic neurons [8]. However, except in a few specific brain regions, such as the dorsal raphe and tuberomammillary nucleus, MAOB is exclusively expressed within astrocytes in most brain regions [8,9,10]. Therefore, the physiological role of MAOB in the brain is most likely attributed to astrocytes.

Interestingly, MAOB expression is clearly increased with aging in most structures of rodent brains, including the cortex, hippocampus, and striatum [11,12]. This age-related increase in MAOB starts right after birth and continues for more than one year [11,12]. Furthermore, human brain tissue shows a delayed but significant age-related increase in MAOB, which starts at the age of 50–60 years [13]. Another study using molecular imaging of MAOB with [^11^C] L-deprenyl-D2 and positron emission tomography (PET) imaging in the living human brains demonstrated that MAOB level was increased in all brain regions examined, except the cingulate gyrus, along with aging [14]. Based on the exclusive expression of MAOB in astrocytes, the age-related increase in MAOB expression in astrocytes could contribute to brain aging.

In addition, MAOB expression is significantly increased in the neuroinflammatory and neurodegenerative conditions, which is associated with reactive astrogliosis [15,16,17,18,19,20]. For example, several studies using various techniques, including immunohistochemistry, enzyme activity assay, and molecular PET imaging, have shown that MAOB expression is significantly increased in the reactive astrocytes of the hippocampus and frontal cortex of the brains with Alzheimer’s disease (AD) [15,16,17,19,20]. In Parkinson’s disease (PD), MAOB expression is significantly increased in the reactive astrocytes of the substantia nigra pars compacta (SNpc) [18,21,22]. These emerging lines of evidence have suggested that the increased MAOB expression along with reactive astrogliosis could contribute to the progression and maintenance of neuroinflammation and neurodegeneration.

## 2. Challenges for Defining the Role of MAOB in Parkinson’s Disease

PD is the most prevalent neurodegenerative motor disorder, which is caused by nigrostriatal DA depletion [23]. It is characterized by remarkable motor dysfunction, including bradykinesia, resting tremor, gait disturbance, and postural instability [24]. Apart from levodopa, which is the most popular therapeutic agent for PD, several irreversible MAOB inhibitors have been widely prescribed for PD patients as an early monotherapy or an add-on to other medications, despite some reports demonstrating their discouraging clinical effects [25,26].

There are two isoenzymes of MAO: MAOA and MAOB. While both isoenzymes are mitochondria-bound flavoenzymes [27], their physiological and pathological roles are distinct due to their differential cellular localization [28] and distinct substrate selectivity. First, MAOA is mainly localized in the nigrostriatal DAergic axon terminals [28,29,30], whereas MAOB is located exclusively in astrocytes and serotonergic neurons [29,30]. Second, they have distinct molecular differences in enzymatic properties revealed by cDNA cloning and peptide sequencing of human MAOA and MAOB [31,32], allowing their differential substrate selectivity. In particular, MAOA is primarily responsible for the metabolism of epinephrine, norepinephrine, melatonin, and serotonin, whereas MAOB is responsible for the degradation of phenylethylamine and benzylamine [33]. Notably, DA is traditionally known to be degraded by both MAOA and MAOB. Particularly, MAOB is known to be upregulated in PD patients’ brains [34]. Therefore, MAOB has long been believed to contribute to PD pathophysiology through excessive DA degradation [35]. This belief was further supported by a positive but limited clinical efficacy of irreversible MAOB inhibitors such as selegiline and rasagiline for PD patients [36,37].

Since 2014, several investigations have begun to emphasize the specific involvement of MAOB, but not MAOA, in astrocytic GABA production via the putrescine degradation route [15,16,21,38,39,40], which has been overlooked in the brain for decades. Moreover, MAOB has recently received special attention due to its increased level along with reactive astrogliosis in neurodegenerative diseases, including PD [15,16,17,18,21,22,38,41]. These recent discoveries on the pathological role of astrocytic MAOB led us to revisit the MAOB’s role in PD pathophysiology. The therapeutic efficacy of MAOB inhibitors for PD is not attributed to a blockade of DA degradation. Instead, it is attributed to a blockade of the excessive astrocytic GABA synthesis and the aberrant tonic inhibition of DAergic neurons in the SNpc. In this review, we summarize the traditional views and recent perspectives in an attempt to redefine the pathological role of MAOB in PD pathophysiology.

### 2.1. Traditional Views on MAOB as a DA-Metabolizing Enzyme

As described above, MAOA and MAOB have differential substrate selectivity. In particular, epinephrine, norepinephrine, melatonin, and serotonin are known to be metabolized by MAOA, whereas phenylethylamine and benzylamine are known to be degraded by MAOB [33]. Among several various monoamines, DA has been one of the most well-known substrates for MAO for the past six decades ago [42]. Since the first-generation specific inhibitors of MAOA and MAOB, clorgiline, and selegiline, respectively, were developed in the 1960s, researchers started to investigate which isoenzyme was responsible for DA metabolism in the 1970s. Several in vivo studies with selective inhibitors against MAOA and MAOB demonstrated that DA is much preferentially degraded by MAOA compared to MAOB in the rat brain [43,44]. On the other hand, some other studies using selegiline also demonstrated that MAOB catalyzes DA as a substrate in rats [45,46,47]. These conflicting results could be partially attributed to the low selectivity of selegiline to MAOB over MAOA (~150-fold difference). Indeed, the affinity of DA deamination was 2.5-fold higher to MAOA than MAOB [47]. Notably, an investigation with the homogenates of brain tissues from various species by Garrick and Murphy demonstrated that DA was found to be deaminated largely by MAOA in the rat brains, whereas by MAOB in the brains of humans and vervet [46]. However, this pivotal finding was limited by the fact that the enzymatic activities of MAOA and MAOB were measured with tissue homogenates which could be different from the intact brain. Despite the controversies on the contributions of MAOA and MAOB to DA degradation, it has been traditionally and until now generally believed that both MAOA and MAOB are equally active towards DA degradation [35,48,49,50,51].

**Figure 1:**
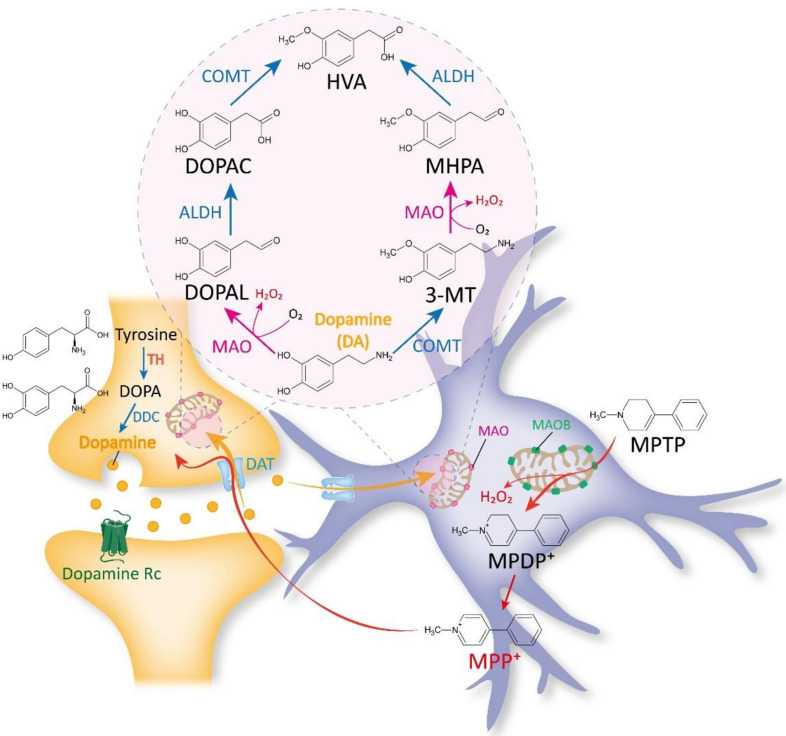
The traditional view on MAOB as a DA- and MPTP-metabolizing enzyme. MAOA and MAOB have been traditionally believed to be responsible for dopamine (DA) metabolism. DA taken up via DA transporter (DAT) is known to be metabolized into homovanillic acid (HVA) through two different pathways. First, MAO converts DA to 3,4-dihydroxyphenylacetaldehyde (DOPAL), which is then converted to 3,4-dihydroxyphenylacetic acid (DOPAC) by aldehyde dehydrogenases (ALDH). DOPAC is finally converted to HVA through catechol-o-methyltransferase (COMT). Second, COMT converts DA to 3-methoxytyramine (3-MT), which is then converted to 3-methoxy-4-hydroxyphenylacetaldehyde (MHPA) by MAO. Then, MHPA is finally converted to HVA by ALDH. Due to the presence of these enzymes in both DAergic presynaptic terminals and astrocytes, it has been assumed that DA is degraded by MAOA and MAOB in both cell types.

Once DA is released from the DAergic presynaptic terminals, it binds to and activates DA receptors. After the receptor activation, DA quickly becomes unbound from its receptors and is absorbed back into the presynaptic neurons through the DA transporter (DAT) [52]. There are two main metabolic pathways for degrading DA. First, MAO catalyzes the conversion of DA to 3,4-dihydroxyphenylacetaldehyde (DOPAL), which is, in turn, metabolized into 3,4-dihydroxyphenylacetic acid (DOPAC) by aldehyde dehydrogenase (ALDH). Finally, DOPAC is decomposed into an inert metabolite homovanillic acid (HVA) by catechol-o-methyltransferase (COMT) [53,54]. Second, COMT catalyzes the conversion of DA to 3-methoxytyramine (3-MT), which is, in turn, metabolized into 3-methoxy-4-hydroxyphenylacetaldehyde (MHPA) by MAO. Finally, MHPA is also decomposed into the final product HVA via ALDH [53,54] (Figure 1). The enzymes in the DA-degradation pathway are expressed in both DAergic presynaptic neurons and astrocytes. However, DATs are differentially expressed in DAergic presynaptic neurons and astrocytes: high in neurons but low in astrocytes [55,56]. This paradoxical low expression of DAT in the astrocytes of the nigrostriatal DA pathway [55,56] has implicated the limited participation of astrocytes in DA degradation in various brain regions, including the striatum, nucleus accumbens, and substantia nigra.

DA degradation is particularly important in the pathophysiology of PD, in which DA deficiency is the key etiology. Among the DA-degrading enzymes, MAOB expression has been reported to be increased in PD patients, while MAOA expression has not [57,58,59]. Therefore, the augmented MAOB-mediated DA degradation has been accepted to contribute to PD pathology [51]. Moreover, H_2_O_2_ is believed to be produced during the MAOB-mediated DA degradation, which could also exacerbate DAergic neuronal dysfunction and degeneration in PD [34,59]. These findings have been somewhat supported by a positive but limited clinical efficacy of irreversible MAOB inhibitors such as selegiline and rasagiline [36,37,60], while the controversies on whether MAOB is responsible for PD pathology through DA degradation in vivo have been left unresolved.

### 2.2. Past and Recent Discoveries against the Belief of MAOB as a DA-Degrading Enzyme

In rodents, accumulating lines of evidence have demonstrated that MAOB is not engaged in regulating DA levels in vivo. Fornai et al. showed that there was no difference in striatal DA levels between MAOB-deficient and wild-type mice [61], implicating that MAOB does not contribute to DA metabolism in the striatum. Consistently, the administration of MAOB inhibitors such as selegiline has been reported to be unable to alter the efflux of DA in the striatum and nucleus accumbens of rats [62,63,64]. On the other hand, acute pharmacological inhibition of MAOA with clorgiline reduced DA metabolism [62,64]. Moreover, another study reported that it was not MAOB inhibition but MAOA inhibition that dramatically blocked DA degradation in rat models of PD [65]. These results imply that it is not MAOB but MAOA which contributes to DA metabolism. Furthermore, people deficient in the MAOB gene show no apparent phenotypes, whereas people deficient in the MAOA gene display severe mental retardation and psychological defects [66], suggesting that MAOB is less likely to participate in DA degradation in humans.

In support of these previous reports, a recent report, employing the latest technologies, provided more compelling lines of evidence demonstrating that not MAOB but MAOA is critically engaged in DA degradation, at least in rodents [39]. Cho et al., utilized fast cyclic voltammetry (FSCV) and multiple trains of five cyclic square wave voltammetry (M-CSWV) to electrochemically monitor the phasic and tonic DA currents evoked by electrical stimulation of the nigrostriatal pathway in the in vivo rat striatum, respectively. Both phasic and basal DA levels were only affected by MAOA inhibition but not by MAOB inhibition, indicating that not MAOB but MAOA contributes to in vivo DA metabolism [39]. This study also adopted a newly developed potent, reversible, and selective MAOB inhibitor, KDS2010 [16], which could circumvent the limitations of previous studies using the irreversible MAOB inhibitor, selegiline. These findings were also confirmed by ex vivo DA imaging using the latest GRABDA2m sensor [39].

The contribution of MAOB to DA degradation in the brain could be ascribed to the difference in the experimental conditions, depending on whether an experiment is performed in vitro or in vivo. Several in vitro studies demonstrated that MAOB also utilizes DA as a substrate in both rats (Km = ~340 μM) [67] and humans (Km = ~210–230 μM) [68], indicating the capability of MAOB in DA degradation in vitro. In the living brain, however, DA needs to first enter the astrocytes, where MAOB is mainly localized, for being deaminated by MAOB. However, DAT expression is relatively low in the astrocytes of various brain regions, including the striatum, nucleus accumbens, and SNpc [55,56], whereas DAT is mainly expressed in the DAergic neurons. Consequently, regardless of MAOB’s ability to metabolize DA in vitro, the contribution of astrocytic MAOB to DA degradation is negligible in the living brain. Taken together, the accumulating lines of evidence call for redefining the role of MAOB in the brain by excluding DA metabolism in the PD pathology.

### 2.3. Traditional Views on MAOB as an MPTP-Metabolizing Enzyme

Another traditional view of MAOB’s action originates from an unfortunate accident. The neurotoxicity of MPTP has been known since 1976 when Barry Kidston, a graduate student in chemistry, self-administered MPTP and began to show parkinsonian symptoms within three days. The autopsy of his brain further revealed the disruption of DAergic neurons and the existence of Lewy bodies in the SNpc [69]. Since MPTP was first reported to cause PD in humans [69], fundamental mechanisms have been studied on how MPTP causes toxicity and motor dysfunction in PD. Now the MPTP-induced mouse model is one of the most widely utilized PD models [70].

MPTP has long been recognized to be a non-toxic substance. However, once MPTP enters the brain, it is believed to be metabolized to a toxic cation MPP+ through MAOB in astrocytes [71]. MPP+ is thought to be taken up by DAergic neurons through DAT and destroys them [72] (Figure 1). Moreover, pharmacological blockade of MAOB inhibitors has been well documented to prevent MPTP-induced PD pathology and parkinsonian motor symptoms [36,73]. These findings led researchers to pay attention only to the MAOB’s role in the conversion of MPTP to MPP+ in the MPTP model, while other possible actions of MAOB in the MPTP-mediated PD-like pathology have been overlooked.

On the other hand, in the MPTP mouse model, it was recently demonstrated that the post-treatment regime of selegiline administration, which starts 3 days after MPTP, significantly alleviated MPTP-induced PD-like pathology and motor symptoms [38]. Since MPTP is known to be converted to MPP+ within 3 days [74], any alleviating effect by selegiline should not be attributed to MAOB’s action of MPP+ conversion. This finding suggests that selegiline may act in ways other than blocking the conversion of MPTP to MPP+.

## 3. Recent Discoveries on MAOB as the Astrocytic GABA- and H_2_O_2_-Synthesizing Enzyme

In contrast to the traditional view on the role of MAOB via DA degradation and MPTP conversion in the pathophysiology of PD, MAOB has been demonstrated as a critical enzyme for astrocytic GABA synthesis in several brain areas under physiological conditions, including the cerebellum [40,75] and striatum [39]. Previous reports have revealed that astrocytic GABA is synthesized from putrescine through several steps of enzymatic actions, including MAOB [15,40]. The astrocytic GABA is tonically released through a Ca2+-activated anion channel Bestrophin-1 (Best1) [76] and binds to extrasynaptic GABA_A_ receptors to tonically inhibit the neighboring neuronal activity [21,38,40,76]. The physiological level of MAOB-mediated tonic inhibition varies by brain region, with high levels in the cerebellum and striatum and low levels in the cortex and hippocampus [77]. Notably, MAOA was not responsible for astrocytic GABA-mediated tonic inhibition in the striatum [39]. These new discoveries highlight the distinct role of MAOB, but not MAOA, in the astrocytic GABA synthesis and tonic inhibition of neurons under physiological conditions.

**Figure 2:**
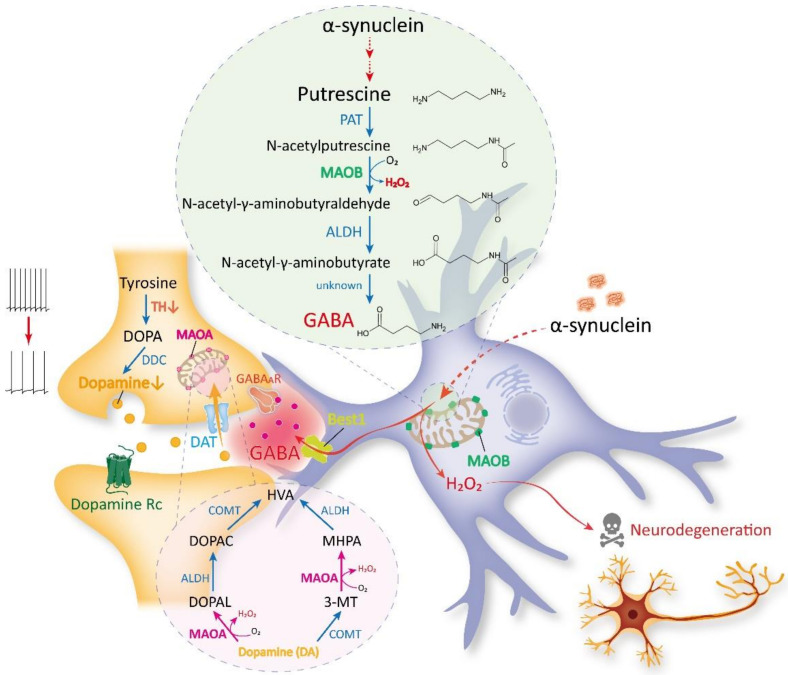
The recent findings on MAOB as a GABA- and H_2_O_2_-synthesizing enzyme. According to recent discoveries, it is not MAOB but MAOA that is responsible for dopamine (DA) metabolism to HVA. Instead, MAOB is responsible for astrocytic GABA and H_2_O_2_ synthesis. In detail, under the PD pathogenesis, the putrescine level in reactive astrocytes is increased, which could be linked to the accumulation of misfolded α-synuclein. Putrescine is converted to N-acetylputrescine by putrescine aminotransferase (PAT). MAOB converts the N-acetylputrescine to N-acetyl-γ-aminobutyraldehyde and produces H_2_O_2_ as a byproduct. N-acetyl-γ-aminobutyraldehyde is sequentially converted to N-acetyl-γ-aminobutyrate and GABA. GABA, released via Bestrophin 1 (BEST1) from astrocytes, inhibits the excitability of neighboring DAergic neurons. On the other hand, H_2_O_2_ exacerbates DAergic neuronal degeneration. The aberrant suppression and deterioration of DAergic neurons lead to reduced expression of tyrosine hydroxylase (TH) and DA deficiency.

Under pathological conditions, including reactive astrogliosis, which is frequently seen in the brains with PD, SNpc reactive astrocytes show high expression of MAOB [21,22,41]. In detail, reactive astrocytes aberrantly synthesize GABA through the enzymatic action of MAOB, leading to excessive tonic inhibition of neighboring DAergic neurons in the SNpc. The aberrant suppression of DAergic neuronal activity leads to a substantial decrease in tyrosine hydroxylase (TH), the key DA-synthesizing enzyme. Notably, the reduction of TH level could be reversible when the DAergic neurons are aberrantly suppressed but still alive. The reduced TH expression causes a deficiency of DA synthesis, which can lead to parkinsonian motor symptoms (Figure 2) [21]. A similar phenomenon of higher MAOB expression and astrocytic GABA in reactive astrocytes has also been reported in the hippocampus of AD brains [15,16,78,79]. These recent discoveries underscore the substantial contribution of astrocytic MAOB in PD pathology through aberrant GABA synthesis.

In addition to GABA, MAOB is critically engaged in H_2_O_2_ synthesis. While MAOB catalyzes the conversion of N-acetylputresicne to N-acetyl-γ-aminobutyraldehyde in the putrescine degradation pathway, H_2_O_2_ is produced (Figure 2). In AD, MAOB-mediated H_2_O_2_ is reported to cause glial activation, tauopathy, neuronal death, brain atrophy, and cognitive impairment [78]. A previous report suggested that astrocytic MAOB-dependent H_2_O_2_ could be critical for PD pathology by demonstrating that MAOB overexpression in astrocytes increased the H_2_O_2_ level and exacerbated the PD pathology [18,80]. This finding was recapitulated by a recent report demonstrating that AAD2004, a potent H_2_O_2_ scavenger, has a significant neuroprotective effect on SNpc of the MPTP mouse model [21]. These reports raise the possibility that MAOB-dependent H_2_O_2_ directly causes DAergic neuronal death in the MPTP model. The astrocytic H_2_O_2_ can exacerbate the astrocytic reactivity and promote neurodegeneration [78], which can largely contribute to PD pathophysiology not only in PD animal models but also in patients. This intriguing possibility of the direct involvement of MAOB-dependent H_2_O_2_ in PD-related neurodegeneration needs to be tested in future investigations.

In this regard, astrocytic MAOB could be the therapeutic target for alleviating PD pathology and the related motor symptoms. This possibility has been tested in the rodent models of PD, including the MPTP mouse model, 6-OHDA rat model, and viral overexpression of A53T-mutated alpha-synuclein mouse model by a pharmacological blockade, gene-silencing, and genetic ablation of MAOB [21,22,41]. MAOB inhibition in these PD models blocked astrocytic GABA synthesis, aberrant tonic inhibition of DAergic neurons, TH expression, and parkinsonian motor symptoms. These recent discoveries delineate the new mode of action of MAOB inhibitors in the treatment of PD as blocking astrocytic GABA and H_2_O_2_ synthesis.

## 4. MAOB as a Therapeutic Target for PD

MAOB inhibitors are categorized into two classes: irreversible inhibitors, including selegiline and rasagiline, and reversible inhibitors, including safinamide and KDS2010 [16]. For the past several decades, the irreversible MAOB inhibitors have been widely utilized in both clinics and animal studies due to their high potency (IC50 = ~10 nM). However, these irreversible inhibitors have a critical drawback which is the low selectivity (selegiline, ~150-fold; rasagiline, ~52-fold) [41], thereby losing their MAOB selectivity at higher doses. The low selectivity of these irreversible inhibitors could have caused the misinterpretation about the role of MAOB in DA degradation because most of the past studies investigating the role of MAOB utilized selegiline and rasagiline.

Also, irreversible inhibitors have limitations as therapeutic agents because their action is short-lived. The long-term treatment (over 2 weeks) of selegiline lost its effect on blocking astrocytic GABA synthesis by turning on a compensatory astrocytic GABA-synthesizing pathway which contains diamine oxidase [15,16]. On the other hand, reversible MAOB inhibitors such as safinamide and KDS2010 do not turn on the compensatory GABA-synthetic mechanism even under the long-term treatment condition.

MAOB inhibitors have been shown to have neuroprotective effects, prevent DAergic neuron degeneration, and decrease parkinsonian symptoms, especially when it is applied to the early phase patients [26,81,82,83,84]. MAOB inhibitors may also reduce the motor fluctuations in PD patients [85]. Up to date, several different MAOB inhibitors are used to treat PD, including Eldepryl or Zelapar (selegiline hydrochloride), Azilect (rasagiline), and Xadago (safinamide). Taken together, reversible MAOB inhibitors can circumvent the drawbacks of conventional irreversible MAOB inhibitors, including low selectivity-induced undesirable effects such as the cheese effect [86] and its short-lived action. Each MAOB inhibitor has been described below.

### 4.1. Selegiline

The first MAOB inhibitor was selegiline, synthesized by Zoltan Ecseri in 1962, patented as an antidepressant in 1965, and developed by Jozsef Knoll as a “psychic energizer [87]”. Selegiline is a selective, irreversible MAOB inhibitor, which is used as monotherapy in the early phase of PD or as a combination therapy with levodopa in more advanced PD states. Selegiline has been shown to delay the time point of levodopa treatment [88,89]. Furthermore, a study of 157 de novo patients suggested that long-term treatment of selegiline delays the progression of the signs and symptoms of PD [90]. However, there is no conclusive evidence from clinical trials to prove that selegiline has disease-modifying effects [91,92,93,94]. Moreover, selegiline has been reported to lose its selectivity to MAOB at high doses (10 mg/kg) [95]. Furthermore, selegiline is metabolized into amphetamine and methamphetamine metabolites, which may cause deleterious effects [96].

### 4.2. Rasagiline

Rasagiline is a second-generation propargylamine-based selective, irreversible MAOB inhibitor. Rasagiline is used as monotherapy to treat early PD or as add-on therapy in more advanced PD. Rasagiline and selegiline are currently licensed in Europe and North America for the symptomatic improvement of early PD. Unlike selegiline, rasagiline has no methamphetamine and amphetamine metabolites. Furthermore, it was shown to have potential anti-apoptotic effects in in vitro and in vivo parkinsonian models [37,60,97]. Although some clinical studies found that rasagiline showed clinical efficacy in both early monotherapy and in advanced PD with levodopa, head-to-head trials comparing rasagiline with selegiline or other inhibitors are needed to test whether rasagiline is superior or not. Furthermore, the FDA label contains warnings that rasagiline may cause severe hypertension or hypotension, worsen the movements in some people, or cause hallucinations [98].

### 4.3. Safinamide

Safinamide is a reversible and selective MAOB inhibitor, approved in March 2017 by the US Food and Drug Administration (FDA) for the treatment of PD patients. Safinamide has undesirable properties in that it also inhibits voltage-gated sodium and N-type calcium channels, modulating glutamate release [73]. It is shown that safinamide has neuroprotective properties thought to be related to MAOB inhibition and ion channel blocking activity [99]. Safinamide was generally well tolerated in clinical trials, but dyskinesia was the most common adverse event [100]. Despite the advantage of being a reversible inhibitor, safinamide suffers from several off-target effects and some adverse effects [101].

### 4.4. KDS2010

A recently developed KDS2010, which is ~12,500-fold more selective to MAOB than MAOA, differentiates the role of MAOB from MAOA and reports that MAOB does not contribute to DA degradation [39]. KDS2010 is a potent (IC50 = 7.6 nM), and selective MAOB inhibitor named shows no known off-target effect (no other enzymes or channels causing >40% inhibition) or toxicity for 4 weeks of repeated dosing in non-human primates [16,41]. KDS2010 was turned out to be highly effective for alleviating the PD-related motor symptoms and PD-like pathology, including reactive astrogliosis, excessive astrocytic GABA, and nigrostriatal DAergic neuronal loss in multiple rodent models of PD [41]. Its clinical efficacy is still waiting to be tested in future studies.

In addition to pharmacology, genetic ablation or silencing of astrocytic MAOB has been reported to alleviate parkinsonian motor symptoms [21,22]. Since MAOB deficiency does not exhibit any abnormalities in humans [66], MAOB might also be used as a safe target for genetic manipulation in the treatment of PD. Recent studies have developed several technologies for gene therapy, including small-interfering RNA (siRNA), short-hairpin RNA (shRNA), an antisense oligonucleotide (ASO), and CRISPR-Cas9, which could be delivered in vivo by an adeno-associated virus (AAV) or nanoparticles [102,103,104]. These genetic manipulations may have a one-off potential for long-term benefits. The genetic ablation of MAOB using these approaches might be considered a promising next-generation therapeutic strategy for early PD patients in the future.

## 5. Concluding Remarks

In this review, we have comprehensively reviewed how recognition of the MAOB’s role in PD pathology has evolved over time, from its traditional role as a DA-degrading enzyme to the recent findings as a critical enzyme regulating astrocytic reactivity. Accumulating lines of evidence have indicated that MAOA, not MAOB, is mainly responsible for mediating DA degradation. On the other hand, MAOB is responsible for the aberrant suppression and deterioration of DAergic neurons through excessive GABA and H_2_O_2_ synthesis in reactive astrocytes of the rodent PD models. A better understanding of the pathogenic role of MAOB in PD will aid in better deciding when MAOB inhibitors are used to improve the efficacy. Furthermore, redefining the MAOB’s role and the mode of action of MAOB inhibitors will spur the new development of innovative therapeutic techniques to fine-control the MAOB’s activity or gene expression.
